# White Spot Lesions (WSLs)—Post-Orthodontic Occurrence, Management and Treatment Alternatives: A Narrative Review

**DOI:** 10.3390/jcm12051908

**Published:** 2023-02-28

**Authors:** Luminita Lazar, Alexandru Vlasa, Liana Beresescu, Anamaria Bud, Ana Petra Lazar, Larisa Matei, Eugen Bud

**Affiliations:** 1 Faculty of Dental Medicine, University of Medicine and Pharmacy, Science and Technology George Emil Palade, 540139 Târgu-Mureș, Romania; 2 Department of Oral and Maxillo-Facial Surgery, Clinical Hospital of Târgu-Mureș, 540139 Târgu-Mureș, Romania

**Keywords:** enamel demineralization, white spot lesion, orthodontic treatment, caries, dental decay, dental cavities

## Abstract

Although treatment with fixed or mobile appliances has become an important part of modern orthodontics, side effects such as white spot lesions (WSLs) have a negative impact on the aesthetic outcome of orthodontic treatment. The purpose of this article was to review current evidence on the diagnosis, risk assessment, prevention, management and post-orthodontic treatment of these lesions. Data collection was performed electronically, and the initial search using the keywords “white spot lesions”, “orthodontics”, “WSL”, “enamel” and “demineralization” in different combinations resulted in 1032 articles for the two electronic databases used. Ultimately, a total of 47 manuscripts were considered relevant to the aim of this research and included in this review. The results of the review indicate that WSLs remain a significant problem during orthodontic treatment. According to studies in the literature, the severity of WSLs correlates to the duration of treatment. Using toothpaste with more than 1000 ppm fluoride at home reduces the frequency of WSL separation and regular application of varnishes in the office reduces the frequency of the occurrence of WSLs only in the context of maintaining a strict hygiene regime. The old hypothesis that elastomeric ligatures retain more dental plaque than metal ones has been refuted. There are no differences in the appearance of WSLs between conventional brackets and self-ligating brackets. Clear aligner mobile devices develop fewer WSLs but are more extensive as opposed to conventional fixed devices, while lingual orthodontic appliances have a lower incidence of WSLs, and the most effective device for preventing these lesions is WIN, followed by Incognito.

## 1. Introduction

Orthodontic treatments have enjoyed growing popularity in recent years, and the white chalky spots, also called white spot lesions (WSLs), that appear after treatment are a major problem in dental aesthetics [1]. There are several causes of enamel demineralization, including poor nutrition and hygiene, and the use of incorrect adhesive techniques [2]. Although treatment with fixed or mobile appliances has become an important part of modern orthodontics, side effects such as WSLs have a negative impact on the aesthetic outcome of orthodontic treatment. The reported prevalence varies in each study [2,3,4,5,6], from 97%, as reported by Boersma et al. [5], to 33.8%, as reported by Geiger et al. [6], depending on the method/criteria of analysis chosen and whether the pre-existing enamel lesions are or are not included the reference unit used (surface, tooth or patient). In some cases, these demineralization lesions may be reversible; the chalky appearance may be partially neutralized by salivary proteins that remineralize the enamel surface [7]. However, in the case of orthodontic treatments, these lesions of the enamel evolve progressively and become irreversible, leading to carious processes [8]. Clinically, enamel demineralization initially occurs through white chalky spots, which also occur in dental fluorosis. In contrast, demineralization presents a high risk of developing tooth decay due to the friability of the enamel and the creation of retentive areas for bacterial plaque [9,10]. Demineralization is the process of removing mineral ions from hydroxy-apatite (HA) crystals in hard tissues, such as enamel, dentin, cement and bone. The restoration of these mineral ions in HA crystals is called remineralization. Both processes take place on the surface of the teeth, and a substantial number of mineral ions in HA can be lost without destroying their integrity, but high sensitivity to heat, cold, pressure and chemical stimuli would be expected [11,12]. Demineralization is a reversible process; therefore, partially demineralized HA crystals may increase to their original size if exposed to an oral environment that favors remineralization [13]. Chemical demineralization of teeth is caused by acid attack via two main means: food acid consumed by food or drink and microbial attack by bacteria present in the oral cavity [14].

However, each has its own specific disadvantages. Most importantly, orthodontic appliances, especially brackets, ligatures and arches, create new retentive areas with the undesired effect of plaque buildup [15]. An increase in the amount of dental plaque containing cariogenic bacteria is the main etiological factor in decalcifying the enamel during orthodontic treatment. This demineralization of the dental surfaces results in the appearance of white spots (WSLs) or even caries. However, in the literature, there are contradictory results on the relationship between orthodontic treatment and the development of dental caries [5,16,17]. Many preventive methods have been defined, such as the topical application of fluoride, the use of fluoride-releasing binders, the use of sodium fluoride mouthwashes and the application of chlorhexidine [18,19,20]. Providing adequate oral hygiene has a significant role in preventing demineralization and thus the formation of carious processes during orthodontic treatment [20]. Furthermore, fixed bonded retainers seem to be a good option for preventing tooth relapse after Invisalign and conventional fixed treatments; therefore, there is also an increased risk of developing WSLs after orthodontic treatment [21]. Genetic, prenatal, perinatal and postnatal factors, such as the breastfeeding period (2%), asthma (16%), high-fever episodes (20%), infections/illnesses (20%), chickenpox (12%), antibiotic intake (8%), diarrhea (4%) and pneumonia (4%) are also reported to influence dental demineralization processes [22]. Although WSLs are one of the most frequent and obvious side effects of orthodontic treatment, the effectiveness of WSL interventions has not yet been adequately evaluated in evidence-based medicine [23,24].

Due to the fact that there are many conflicting opinions regarding the etiology of demineralization of enamel after orthodontic treatment, the purpose of this article was to review current evidence on the management of WSLs and post-orthodontic treatment alternatives.

## 2. Materials and Methods

### 2.1. Eligibility Criteria

Inclusion criteria:

- Scientific articles published from 1 January 2010 to 31 December 2022;
- Scientific articles published in the English language;
- Clinical studies that mention development of WSLs during or after orthodontic treatment:
  - The literature search was not restricted to any age interval, sex, duration of treatment or orthodontic disharmony treated with fixed appliances.
  - Only articles related to the development of WSLs in connection with orthodontic treatment were considered relevant to the purpose of this review.

Exclusion criteria:

- Papers with no clear report of clinical study
- Lesions that are not present in the oral cavity or in a well-specified location in title and/or abstract;
- Animal studies;
- In vitro studies, case reports and case series.

### 2.2. Literature Search Strategy

#### 2.2.1. Data Sources

**Figure 1:**
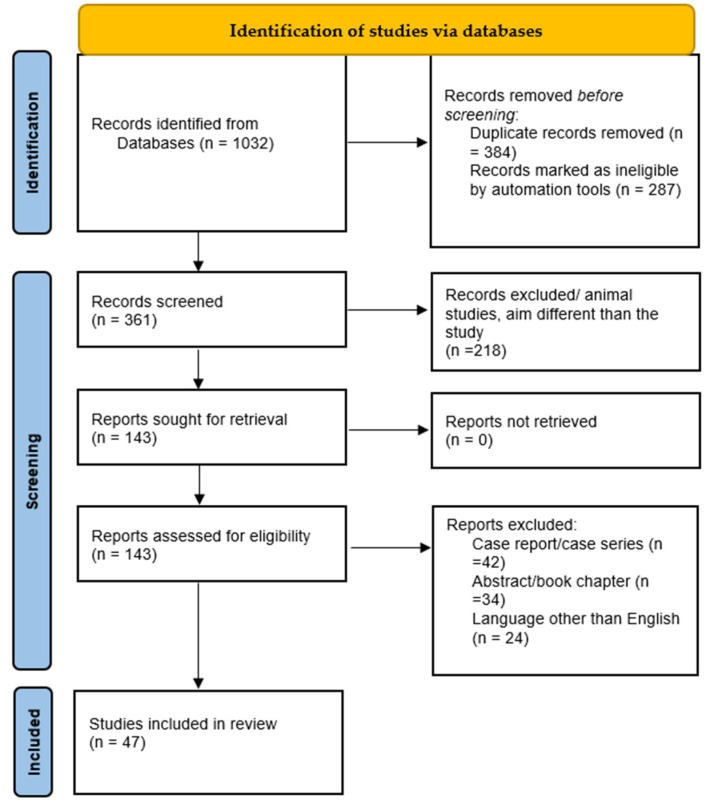
PRISMA flow chart representation of the methodology for the conducted study.

In order to have a broader view of the subject, with the aid of the PubMed Central electronic library and Cochrane database, a comprehensive manual search was conducted. The main search was performed up to 31 December 2022, using the keywords “white spot lesions”, “orthodontics”, “WSL”, “enamel” and “demineralization” in different combinations (Figure 1). For quantification of the search results, the PRISMA flow chart guidelines [25] was used.

#### 2.2.2. The Data Collection Protocol Used in This Review Was as Follows

Data collection was performed electronically, and the initial search using the keywords “white spot lesions”, “orthodontics”, “WSL”, “enamel” and “demineralization” in different combinations resulted in 1032 articles for the two electronic databases used. To narrow down the number of articles and remove articles that were not of interest for our study, the following procedures were performed:

- Selection of items from the Dentistry and Oral Health category;
- The population was not restricted in any way (any race, sex, age, geographical location);
- Selection of studies that included only orthodontically treated abnormalities by fixed or mobile methods;
- Selection of articles only provided as a full text.

A total of 1032 documents in all were found following a thorough search of the online journals. Similar/duplicate publications were removed, leaving 143 distinct papers that were initially available. After reviewing the submissions’ abstracts and titles, 96 more articles were disqualified. The abstracts of the studies to be included were manually analyzed by two authors; the titles and abstracts of retrieved studies were screened and all the studies that contained one or more of the exclusion criteria were excluded. The articles selected for full-text reading were examined by the two authors, and those that were lacking relevant information for the purpose of this review were excluded. Any controversy was resolved with the aid of a third reviewer, selected among the authors. Ultimately, 47 documents that satisfied the necessary inclusion and exclusion criteria were selected.

### 2.3. Risk of Bias

In order to evaluate the methodological quality of included studies, the articles’ data were independently evaluated by the authors using a special manual form designed according to the following categories: study model design, major outcomes related to WSLs associated with orthodontic appliances, number of subjects and study results.

## 3. Results

This paper includes scientific articles published in the English language in recent years and clinical studies exploring orthodontic treatment and WSL occurrence and evolution related with different conditions. The results are systematized as follows.

### 3.1. Results According to the Prevention Method Used

#### 3.1.1. Toothpaste with Fluoride

**Figure 2:**
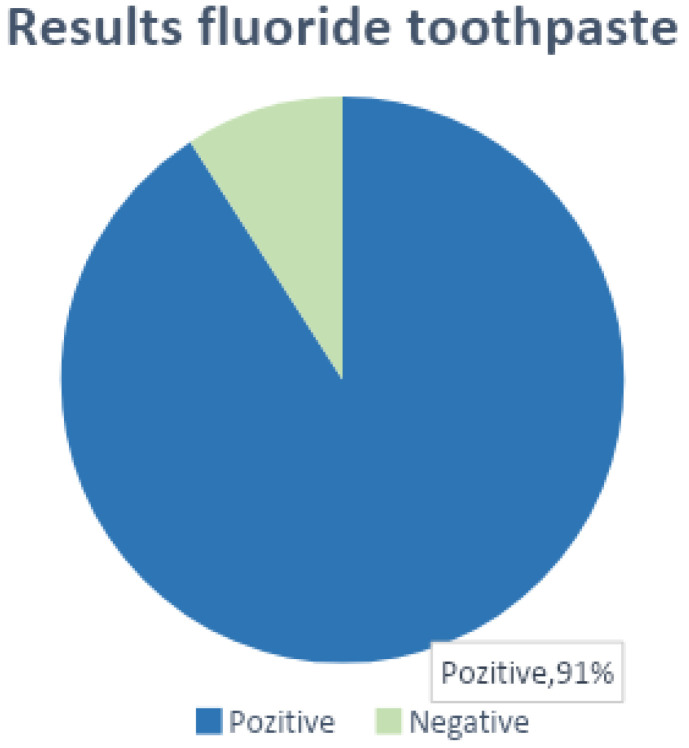
Results that confirm/refute the effectiveness of fluoride pastes in WSL prevention.

One of the most common topics in the selected articles was the effectiveness of high-fluoride toothpastes for WSL prevention (Table 1, Figure 2). In general, the concentration of sodium mono-fluorophosphate found in a common brand of toothpaste was 0.76% and the concentration of sodium fluoride was 0.22%. It was shown that the use of toothpaste with a fluoride concentration (in one of these forms) of over 1000 ppm reduces the frequency of enamel lesions by 20% [24].

Of the 47 studies analyzed, 10 of them (21.3%) showed that the use of toothpaste with a high fluoride concentration at home or in combination with the use of fluoride toothpastes in the office periodically leads to satisfactory results in preventing the occurrence of WSLs. One study did not find conclusive differences between the studied group and the control group, and 34 of the studies did not specify this aspect. A total of 91% had positive results, demonstrating the usefulness of fluoride toothpaste in the prevention of WSL.

#### 3.1.2. Varnish with Fluoride

**Figure 3:**
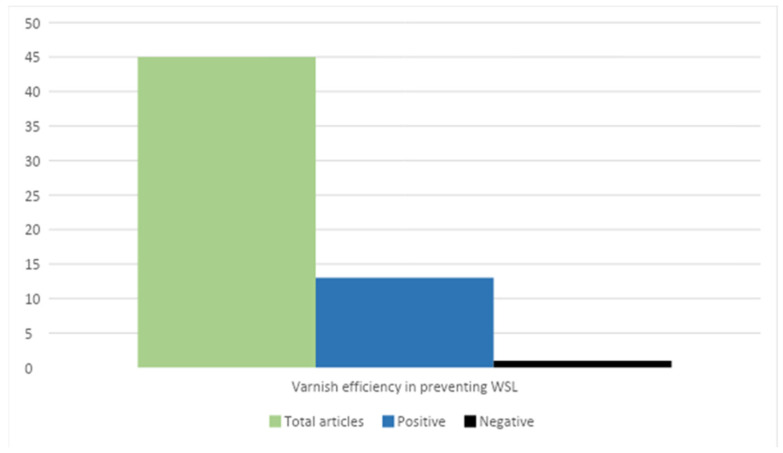
The effectiveness of varnish-type varnishes in the prevention and treatment of incipient WSL lesions. Positive results: Restrepo M et al. [29], Rechmann P et al. [30], Federico Perrini et al. [35], Ashok Kumar Jena et al. [36], Nicoline CW van der Kaaij et al. [46], Adílis Kalina Alexandria et al. [49], Lídia Lipták et al. [50], Roslyn J Mayne et al. [61], Benson PE et al. [64], Gamze Metin-Gürsoy et al. [70], Marco Aurélio Paschoal et al. [57]. Negative result: Marcela Cristina Damião Andrucioli et al. [55].

Using only standard prophylaxis, the treatment of demineralization around brackets has progressed. Additional prophylactic measures such as fluoride varnishes are needed. Among the analyzed studies that address the use of fluorinated varnishes and gels in the office for the prevention of WSLs and even the treatment of incipient lesions, 14 of the studies demonstrated the effectiveness of varnishes for this purpose (Figure 3).

Of the 14 articles evaluating the effectiveness of varnishes, only 1 study showed negative results. In their study, Damião Andrucioli et al. [55] compared the levels of *Streptococcus mutans* (MS) in saliva and the retained bacterial plaque around the brackets. In study group 1, a resin-modified glass ionomer cement (RMGIC) was used for cementing the brackets, and for group 2, a composite resin was utilized. Cementing with the RMGIC did not influence the number of MSs adjacent to the brackets; instead, the composite resin showed a progressive increase in MSs, with the appearance of demineralization signs. Topical applications with fluoride were not clinically relevant in this situation. The MS level was not altered in any of the groups following acid demineralization. Their conclusion suggested that topical application with fluoride has not been shown to be effective in reducing MSs and thus subsequent demineralization.

#### 3.1.3. Differences between Paste and Varnish

Rechmann P, Bekmezian S et al. [30] attempted to demonstrate the difference in efficacy between fluoridated pastes and fluoridated varnish application in a study of 37 patients undergoing orthodontic treatment for 12 months. A paste with a concentration of 1100 ppm fluoride and a varnish were used once every 4 days in their study, for the experimental group and the control group. The same paste, with 1100 ppm fluoride, was used, but the varnish was replaced with mouthwash with a high concentration of fluoride. No statistically significant differences were found in the EDI (Early Development Index) or ICDAS (International Caries Detection and Assessment System). Salivary fluoride levels were significantly higher at 12 months for the experimental group than for the control group (0.20 ± 0.26 vs. 0.04 ± 0.04 ppm), demonstrating the superior efficacy of topical varnish applications used clinically [47].

#### 3.1.4. Use of Probiotics

Numerous studies have shown how probiotics act to limit the proliferation of certain acidogenic bacteria, which motivated an attempt to demonstrate probiotics’ effect on the prevention of WSLs. This acid-neutralizing property was tested on 60 patients with fixed orthodontic appliances by Jose JE et al. [71]. They were divided into three groups of 20: the first group was the control, group 2 followed treatment with oral probiotics, and group 3 used toothpaste with probiotics daily.

The plaque index was analyzed twice: before the start of the study and after 30 days. The presence of *Streptococcus mutans* was assessed using the real-time PCR technique. The statistical analysis showed that there were significant reductions in the concentration of S mutans in groups 2 and 3 compared to group 1, but there were no statistically significant differences between groups 2 and 3. This study demonstrated the superior efficacy of probiotic toothpaste over oral probiotics in preventing WSLs.

On the other hand, Sotiria Gizani et al. [44] denies the effectiveness of using probiotics for WSL prevention, based on the results of a study of 85 patients. The study period was between 7 and 24 months after the removal of the orthodontic device. They were randomly assigned to the test group or placebo group. Subjects in the test group were instructed to take a probiotic pill containing two strains of *Lactobacillus Reuteri* once a day. Dental plaque, WSLs and salivary MS and LB levels were recorded at baseline and immediately after orthodontic device removal. This study concluded that the intake of probiotics has no clinical influence on the development of orthodontically induced WSLs with fixed appliances.

### 3.2. Results According to the Type of Ligatures Used (Elastic/Wire)

To identify the relationship between the amount of plaque and pathogenic bacteria present at the edge of the orthodontic appliance and the type of ligature material used, Tyson Buck, Peter Pellegrini et al. [37] analyzed the formation of white chalky spots via photographic evaluation and a fluorescent laser light (DIAGNOdent™). The study was performed on 13 subjects, including wearers of classic orthodontic appliances with elastic ligatures and wearers of SL (self-ligating) brackets. Bacterial plaque samples collected one year after device adhesion were analyzed via ATP bioluminescence, and WSLs were identified with DIAGNOdent™.

Through this study and with the help of ATP bioluminescence, the theory that elastomeric ligatures retain a richer bacterial colony than metal ones, which is upheld by many authors, was refuted. The amount of plaque and colonized bacteria were not significantly different between the two groups. Based on photographic determinations and DIAGNOdent™, WSLs were found to be equally prevalent in the two groups evaluated.

Fatma Deniz Uzuner, Emine Kaygisiz et al. [72] evaluated the effect of the type of ligatures used (conventional brackets with elastic ligatures versus self-ligating brackets) on the levels of *Streptococcus Mutans* (SM) and *Lactobacillus* (LB) in saliva and bacterial plaque. Their study included 40 non-smoking patients aged 14–16 that did not suffer from systemic diseases and were not under any antibiotic or probiotic treatments. They were randomly divided into two groups: conventional treatment (CB) and self-ligating brackets (SLBs). Using the Dentocult™ LB and MS system, no differences were observed in the number of MSs or LBs in the bacterial plaque between groups.

### 3.3. Results Depending on the Type of Orthodontic Appliance Used

#### 3.3.1. Mobile versus Fixed Device

The emergence of alignment orthodontic appliances is considered revolutionary in orthodontics, mainly due to their aesthetic properties. Ziad Albhaisi et al. [38], aiming to analyze the relationship between orthodontic treatment with an aligner (CA—clear aligner) and the development of WSLs with a comparison with fixed orthodontic treatment (FA), conducted a randomized clinical study on 49 patients. CA treatment was performed for group 1 and FA for group 2. WSLs were analyzed using QLF (quantitative light-induced fluorescence). The mean fluorescence loss was 0.4% for the CA group (*p* = 0.283) and 1.2% for the FA group (*p* = 0.013). The average increase in lesion area was 82.2 pixels for the CA group (*p* < 0.001) and 9.3 pixels for the FA group (*p* = 0.225). Additionally, the mean value of newly developed lesions per patient was 6 WSLs in the CA group and 8.25 in the FA group (*p* = 0.039).

Demineralization was present in both groups treated with a mobile or fixed device, with the main difference being that in the CA group, larger but less developed lesions were found, while the FA group developed several new WSLs with a greater depth but a reduced surface size. Additionally, the plaque index (PI) was significantly higher in the group with fixed orthodontic braces.

#### 3.3.2. Incognito versus WIN

Even if the appearance of WSLs on the vestibular surface of the teeth is not noticed by the patients during the treatment, they become a problem after the removal of the appliance, when the aesthetics are affected. The use of the oral surface of the teeth for the placement of brackets does not only present aesthetic advantages but can even be useful for reducing the frequency of enamel demineralization.

In order to make a comparison between the two types of multi-bracket devices (which differ in terms of design, material and manufacturing technology), the incidence of WSLs in subjects treated with lingual orthodontic appliances was analyzed in two studies. Knösel M et al. [59,62] demonstrated that subjects treated with the WIN device were at a lower risk of developing enamel-decalcifying side effects than those treated with the Incognito device. Both types of lingual devices reduced the frequency of WSLs compared to conventional devices.

### 3.4. Results Depending on the Adhesive Material of the Brackets

#### 3.4.1. Cement

Ahmet Yagci et al. [43] compared three cementing agents’ (glass ionomer cement, compomer and poly-carboxyl cement) influence on the formation of WSLs in patients with circuit breakers. Subsequent white chalky spots were analyzed using the QLF method. Subjects were divided into three groups, including a control group that comprised patients who had never had orthodontic treatment. QLF images taken before and after the rapid maxillary expansion treatment were analyzed with regard to the following parameters: the proportion of fluorescence loss compared to healthy tissue and the maximum loss of fluorescence in the entire lesion. Demineralization was present in all three groups studied, and significantly more so in the treated groups than the control group. Polycarboxylate-cemented teeth developed the most WSLs, followed by glass-ionomer-cemented teeth. Compomers showed the best results, with compomer-cemented teeth developing the fewest WSLs. The benefits of glass ionomers were also presented by Damião Andrucioli et al. [55], who compared the levels of Streptococcus Mutans (SM) in saliva and the biofilm around the cemented resin-modified glass ionomer (RMGIC) and a classical composite resin. Their study showed a significant increase in the level of MS in the biofilm adjacent to the composite resin cast elements, while the RMGIC allowed better control of the number of MSs in the adjacent biofilm [55].

#### 3.4.2. Composite Resins

In fixed orthodontic treatments, the most retentive area for bacterial plaque is around the metal bracket, due to the rough surface structure of the adhesive materials. The free surface energy released by them plays an important role in the adhesion of MSs to these materials. The different properties of materials, such as composites, glass ionomers, compomers and other materials, give them a wide range of indications, but none meet the ideal conditions. Alabdullah MM et al. [31] analyzed the effects of a fluoride-releasing composite on enamel and on the appearance of WSLs. Thirty-four patients were followed for twelve months, half of whom underwent orthodontic treatment with the classic composite material and the other half with the fluoride-releasing composite. The percentage of new WSL lesions increased from 6.3% (after 3 months) to 15% (after 12 months) for the control group and from 3% to 16.3% for the study group. No significant differences between the two groups in terms of lesions over time were found in DIAGNOdent™ examinations or photographic analyses. Alabdullah MM et al. [31] concluded that composite resins do not have the desired effect of preventing demineralization and WSL formation during fixed-brace orthodontic treatment.

#### 3.4.3. Sealers

The application of sealants for the protection of enamel is common in patients with fixed orthodontic appliances; however, the data regarding their in vivo durability are quite unclear. Although there are studies showing a decrease in the frequency of WSLs after the application of a sealing agent, very few studies have examined in vivo changes in sealant integrity and the influence of hygiene and observation time on results. We compared data from four studies that analyzed the effectiveness of sealers in the prevention of WSLs, of which 100% showed positive results. In combination with proper oral hygiene, fluoride-releasing sealants help reduce the frequency of WSLs [51,58,68,70].

Although these studies demonstrate the effectiveness of sealers in the prevention of WSLs, we must also consider their action over time. In vivo studies by Michael Knösel et al. [62] have shown that sealers provide enhanced protection against new WSLs, but do not provide protection throughout treatment, requiring reapplication. The durability of the sealing material can be influenced both by the time elapsed between applications and by the brushing technique or the forces applied, which cause the abrasion of the material. The integrity of the material was assessed using a UV lamp with black light, under which the sealant appears. The results of their study indicate that sealers are very effective in preventing WSLs, but their effectiveness decreases over time, and thus they require reapplication at approximately three and a half month intervals.

### 3.5. Results Depending on the Bonding Agent Used

#### Direct versus Indirect Technique

Aykan Onur Atilla et al. [51], in order to evaluate the level of enamel demineralization in fixed orthodontic treatment depending on the adhesive method used (direct or indirect), used QLF to compare the results of the two techniques. In their study, the percentage of fluorescence loss (ΔF and ΔF max), the degree of demineralization (ΔQ) and the surface area of the lesion (WS area) were determined and compared according to the adhesive technique. The study conducted on 56 patients demonstrated the low frequency of WSLs in the indirect technique. In addition, the increased efficiency of flowable composites has been demonstrated compared to conventional ones in terms of the occurrence of WSLs.

On the other hand, another study conducted by Kübra Yıldırım et al. [46] comparing the effects of the direct and indirect bond technique found no significant differences in the occurrence of WSLs in the two techniques. The indirect technique was significantly faster than the direct technique, and the marginal closure proved to be superior, but the bacterial plaque accumulation rate and WSL formation were not significantly different between the two groups.

### 3.6. Results Depending on the Demineralization Technique

#### 3.6.1. Self-Etching Primers versus Conventional Demineralization

For the past 50 years, acid etching has been an important step in the bonding of orthodontic appliances. Obviously, the mineral component of enamel decreases over time, which makes it more prone to possible damage. In order to reduce the risks of acid etching, so-called self-etching primers have appeared on the market. Hu H et al. [67] evaluated the efficiency of SEPs (self-etching primers) compared to the conventional demineralization technique with orthophosphoric acid. Their study did not find any useful evidence suggesting whether SEPs or conventional acid etching lead to the formation of a smaller number of WSLs or a smaller surface area.

#### 3.6.2. Total Etching versus Partial Etching

In determining whether total or partial etching techniques influence the occurrence of demineralization, Michael Knösel et al. [62] demonstrated that total demineralization of the vestibular surface, in combination with poor hygiene, has unfavorable WSL results. Excess etching over the entire surface, as well as prolonged acid etching time, should be avoided for the prevention of iatrogenic WSLs. Ahmet Yagci et al. [43] compared the two demineralization methods using quantitative fluorescent light (QLF) imaging. Photographs were taken at the beginning of treatment and at 3 (T1) and 6 (T2) months after the start of treatment, as well as at the end of treatment, after de-bracketing (T3).

Although partial etching seemed to be more successful in the first 6 months, in the long run, the results started to stabilize, and the differences between total and partial acid etching were demonstrated to be statistically insignificant. However, the authors concluded that PE (partial etching) is more successful than TE (total etching), as WSL formation was lower in the PE group than in the TE group, in all periods except T2 to T3 [60].

### 3.7. Results by Sex and Age

Michael Knösel et al. [62] also investigated the relevance of different patient selection criteria (age, sex) for the incidence and severity of WSLs or SBLs (under-bracket lesions). The incidence of WSLs in study subjects ≤16 and >16 years of age was analyzed separately, as the hypothesis of increased sensitivity of enamel lesions in pre-adolescents was considered. WSL development has been shown to be significantly increased in pre-adolescents (≤16 years) compared to adolescents (>16 years) and in male subjects, as demonstrated in 630 subjects.

## 4. Discussions

The results of this review indicate that WSLs remain a significant problem during orthodontic treatment. After analyzing all the included articles and comparing them according to the criteria mentioned above, detection of enamel lesions during orthodontic treatment was determined to be a challenge for the clinician [73]. The entire surface of the tooth should be free of plaque and not covered by the gums, and the tooth should be dried very well in order to detect incipient WSLs. In the absence of QLF or DIAGNOdent™, WSLs could be easily overlooked. These assessment methods are essential for the diagnosis of enamel lesions, being an additional aid for their monitoring [74]. Clinical evaluation using photographs is the simplest and the most commonly used method of diagnosis and monitoring [75]. Due to the subjectivity of this method and the low-quality properties, it should be standardized so that it is reproducible. In the absence of these standard parameters, this assessment should be based on quantitative methodology such as DIAGNOdent™ and QLF [74].

Due to individual differences in patient oral hygiene, eating habits and adherence, which play very important roles in the emergence of WSLs, the conclusions of this review cannot be generalized. Thus, additional studies on large patient populations are needed to validate its clinical relevance. Moreover, the oral hygiene status of patients, as assessed via plaque index scores, should be carefully assessed both before and during orthodontic treatment. Despite these limitations of this review due to different diagnostic and monitoring methods used, the lack of standardization of assessment methods and limited number of patients in some studies, a common finding for all included articles was that fluoride-based remineralization agents reduce the incidence of WSLs. It is very well known that fluoride has been the gold standard for remineralization in clinical practice for a few decades, but in recent years, new remineralizing strategies based on the integration of calcium and phosphates at the level of demineralized dental surfaces have been developed. Hydroxyapatite represents one of the most recently discovered remineralization systems and is used in an innovative biomimetic approach that aims to integrate it completely within the enamel structure and ensure its restoration with the same type of substance from which it is formed [76,77]. Due to its ability to repair dental hard tissue, it can protect teeth from erosions, wear and caries [78], while also being an effective desensitizing agent, even better than fluoride [79]. On the other hand, pre-orthodontic treatment with fluoride lacquer or varnish leads to lower bond strengths, which is likely to result in a higher frequency of premature detachment of the bracket [80]. The hydroxyapatite-based toothpaste tested caused a higher reduction in hypersensitivity/pain values than that found with conventional fluoride toothpaste [81]. So, in the future, hydroxyapatite could be helpful for patients with a high risk of enamel demineralization and carious lesions, such as orthodontic patients, and could perhaps become the new gold standard. Very recent studies suggest that resin infiltration and micro-abrasion techniques are comparably effective in reducing the sizes of WSLs, but resin infiltration enjoys an aesthetic advantage over micro-abrasion [82]. Furthermore, Simon et al. (2002), in their study, suggest that both resin infiltration and casein phospho-peptide-amorphous calcium phosphate have desired and durable aesthetic improvements in terms of a reduction in the area as well as the color of post-orthodontic white spot lesions [83].

A total of 7 out of 280 studies on remineralization, micro-abrasion and resin infiltration met the required criteria. WSL treatment generally involves several sessions, and recall intervals should be adjusted for the assessment of preventive interventions and monitoring of initial lesions and possible training in changing the patient’s oral and behavioral hygiene.

According to studies in the literature, the severity of WSLs correlates to the duration of treatment, and the clinical relevance of comparative studies is significant only for those conducted under the same period of orthodontic treatment. Although the number of articles included is quite small, the implication for the field is significant in that it brings a complex systematization of the data present in the literature. Although most patients have received sanitizing instructions, their cooperation and compliance need to be individually considered. Subsequent studies on the occurrence of WSLs after orthodontic treatment would be a real help in supplementing the information found so far in the literature.

### Limitations of this Review

The results of this review can be seen to have certain limitations, namely the rather small number of studies analyzed. The present review augments the reference data in the literature, supporting future research in this field.

## 5. Conclusions

Using toothpaste with more than 1000 ppm fluoride at home reduces the frequency of WSL separation, and regular application of varnishes in the office reduces the frequency of the occurrence of WSLs only in the context where strict hygiene is maintained. The old hypothesis that elastomeric ligatures retain more plaque than metal ones has been refuted. There are no differences in the appearance of WSLs between conventional brackets and self-ligating brackets. Additionally, clear aligner mobile devices develop fewer but more extensive WSLs as opposed to conventional fixed devices, while lingual orthodontic appliances have a lower incidence of WSLs, with the most effective being WIN, followed by Incognito.

## Figures and Tables

**Table 1:** Effectiveness of high-fluoride toothpastes for WSL prevention. X: not specified; +: positive result; -: negative result.

Article	Results
1. Hoffman et al. [26]	+
2. Restrepo et al. [27]	X
3. Sonesson et al. [28]	X
4. Knösel et al. [29]	X
5. Rechmann et al. [30]	+
6. Alabdullah et al. [31]	X
7. Gómez et al. [32]	X
8. Yagci et al. [33]	X
9. Hammad et al. [34]	+
10. Perrini et al. [35]	+
11. Jena et al. [36]	X
12. Buck et al. [37]	X
13. Albhaisi et al. [38]	X
14. Esenlik et al. [39]	+
15. Robertson et al. [40]	+
16. O’Reilly et al. [41]	+
17. Cheng et al. [42]	X
18. Yagci et al. [43]	X
19. Gizani et al. [44]	X
20. van der Kaaij et al. [45]	X
21. Yıldırım et al. [46]	X
22. Kumar et al. [47]	X
23. Adílis et al. [48]	X
24. Quaranta et al. [49]	X
25. Lipták et al. [50]	X
26. Atilla et al. [51]	X
27. Tüfekçi et al. [52]	X
28. Eppright et al. [53]	X
29. Danaei et al. [54]	X
30. Damião Andrucioli et al. [55]	-
31. Benson et al. [56]	+
32. Paschoal et al. [57]	X
33. Kantovitz et al. [58]	X
34. Knösel et al. [59]	X
35. Oosterkamp et al. [60]	X
36. Mayne et al. [61]	X
37. Knösel et al. [62]	X
38. Davari et al. [63]	x
39. Benson et al. [64]	+
40. Millett et al. [65]	X
41. Mandall et al. [66]	X
42. Hu et al. [67]	X
43. Millett et al. [68]	X
44. Pereira-Cenci et al. [69]	X
45. Metin-Gürsoy et al. [70]	+
46. Jose et al. [71]	X
47. Uzuner et al. [72]	X

## Data Availability

Data supporting reported results, including links to publicly archived datasets analyzed can be checked with the corresponding author at alexandru.vlasa@umfst.ro.
